# How Full-Length FVIII Benefits from Its Heterogeneity – Insights into the Role of the B-Domain

**DOI:** 10.1007/s11095-019-2599-2

**Published:** 2019-04-01

**Authors:** Julia Anzengruber, Martin Feichtinger, Philipp Bärnthaler, Norbert Haider, Josenato Ilas, Nina Pruckner, Karima Benamara, Friedrich Scheiflinger, Birgit M. Reipert, Mantas Malisauskas

**Affiliations:** 1 Research & Development, Baxalta Innovations GmbH, a Takeda company, Vienna, Austria; 2 Technical Operations, Baxalta Innovations GmbH, a Takeda company, Vienna, Austria

**Keywords:** blood proteins, factor VIII, haemophilia A, protein aggregates, protein stability

## Abstract

**Purpose:**

To explore how the natural heterogeneity of human coagulation factor VIII (FVIII) and the processing of its B-domain specifically modulate protein aggregation.

**Methods:**

Recombinant FVIII (rFVIII) molecular species containing 70% or 20% B-domain, and B-domain-deleted rFVIII (BDD-rFVIII), were separated from full-length recombinant FVIII (FL-rFVIII). Purified human plasma-derived FVIII (pdFVIII) was used as a comparator. Heterogeneity and aggregation of the various rFVIII molecular species, FL-rFVIII and pdFVIII were analysed by SDS-PAGE, dynamic light scattering, high-performance size-exclusion chromatography and flow cytometry-based particle analysis.

**Results:**

FL-rFVIII and pdFVIII were heterogeneous in nature and demonstrated similar resistance to aggregation under physical stress. Differences were observed between these and among rFVIII molecular species. FVIII molecular species exhibited diverging aggregation pathways dependent on B-domain content. The propensity to form aggregates increased with decreasing proportions of B-domain, whereas the opposite was observed for oligomer formation. Development of cross-β sheet-containing aggregates in BDD-rFVIII induced effective homologous seeding and faster aggregation. Naturally heterogeneous FL-rFVIII and pdFVIII displayed the lowest propensity to aggregate in all experiments.

**Conclusions:**

These results demonstrate that pdFVIII and FL-rFVIII have similar levels of molecular heterogeneity, and suggest that heterogeneity and the B-domain are involved in stabilising FVIII by modulating its aggregation pathway.

**Electronic supplementary material:**

The online version of this article (10.1007/s11095-019-2599-2) contains supplementary material, which is available to authorized users.

## Introduction

Human factor VIII (FVIII) is an essential plasma glycoprotein in the blood coagulation cascade, serving as a co-factor for factor IXa in the conversion of factor X to factor Xa (1,2). A defect or deficiency in FVIII results in haemophilia A, one of the most common severe bleeding disorders (3).

Recombinant protein technology has generated recombinant FVIII (rFVIII) products to treat haemophilia A by protein replacement. Products differ mainly in glycosylation (4) and the presence or absence of the B-domain sequence in the FVIII cDNA, commonly referred to as full-length (FL-) rFVIII and B-domain-deleted (BDD-) rFVIII (5–9).

All protein-based drugs, including FVIII, bear a certain risk to aggregate during manufacturing and shelf storage, and a susceptibility to mishandling during treatment (10,11). In the clinical setting, the presence of aggregates in protein therapeutics has induced unwanted immune responses in some patients, which may affect the therapy’s efficacy (12–20).

**Fig. 1:**
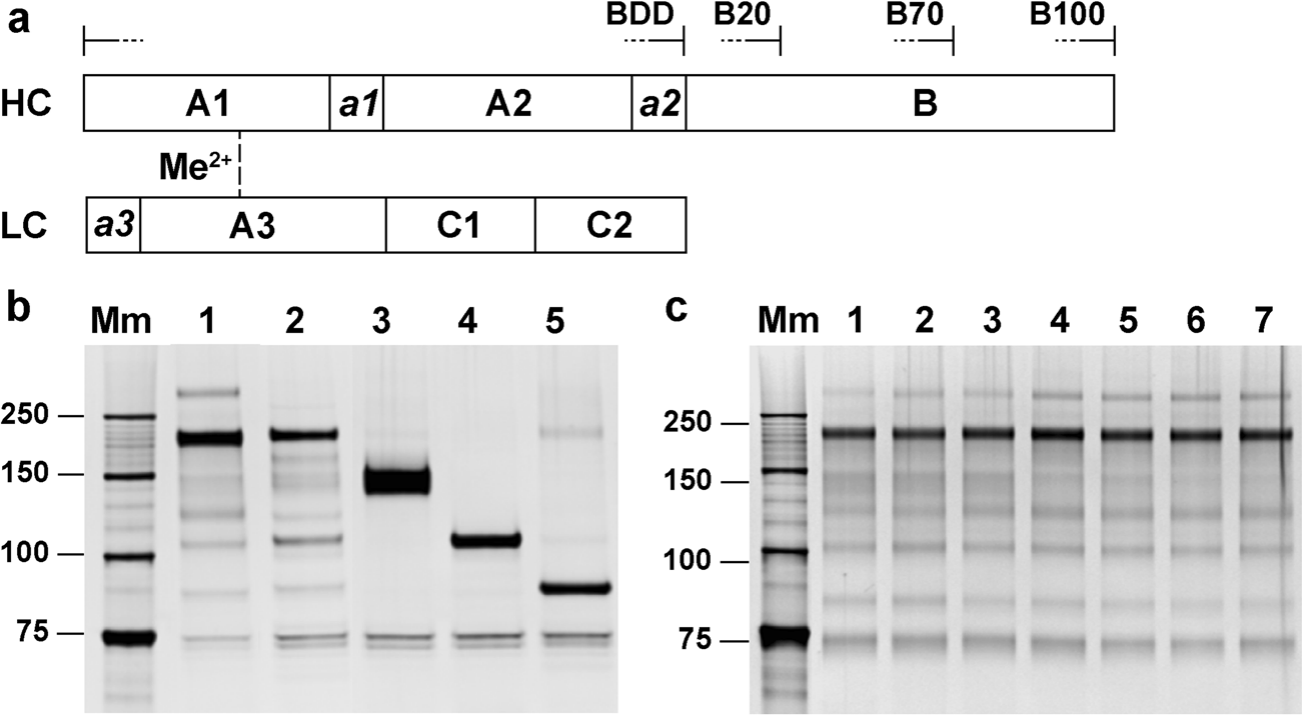
**FVIII heterogeneity and molecular species.** (**a**) Domain structure of FVIII. Brackets indicate major HC/B-domain species present in FL-rFVIII. (**b**) Silver-stained SDS-PAGE gel of FL-rFVIII (1), pdFVIII (2), purified rFVIII species B70-rFVIII (3), B20-rFVIII (4) and BDD-rFVIII (5). (**c**) Silver-stained SDS-PAGE gel of historical lots of FL-rFVIII produced in 2005 (1), 2007 (2), 2008 (3), 2012 (4), 2013 (5), 2014 (6) and 2015 (7). B20-/B70-/B100-rFVIII, human recombinant factor VIII containing 20%/70%/100% B-domain, respectively; BDD-rFVIII, human B-domain-deleted recombinant factor VIII; FL-rFVIII, human full-length cDNA-based recombinant factor VIII; FVIII, factor VIII; HC, heavy chain; LC, light chain; Mm, Precision Plus unstained protein standard (Bio-Rad); pdFVIII, human plasma-derived factor VIII; SDS-PAGE, sodium dodecyl sulfate–polyacrylamide gel electrophoresis.

FVIII is mainly produced by liver sinusoidal endothelial cells (21) as a large single-chain protein comprised of the domain structure NH_2_-A1-a1-A2-a2-B-a3-A3-C1-C2-COOH. Different intra- and extracellular processing of the B-domain causes different heterodimeric molecular species to circulate in plasma. Thus, FVIII contains a constant-sized light chain (LC) (a3-A3-C1-C2) and a heavy chain (HC), minimally composed of the A1-a1-A2-a2 domains, but variable in size due to the presence of some or all of the adjacent B-domain (5) (Fig. 1a). HCs and LCs are associated via a non-covalent linkage that requires a divalent metal ion (22,23).

The FVIII B-domain is heavily glycosylated and although dispensable for procoagulant activity (24), appears to have functional roles throughout FVIII’s lifecycle (25). The B-domain may be involved in intracellular interactions that regulate quality control and secretion (26–28) and in regulation within plasma during activation and clearance (29–31). It has little effect on the overall FVIII secondary structure in solution (32).

We investigated the B-domain’s influence and the natural heterogeneity originating from its presence in FL-rFVIII and human plasma-derived factor VIII (pdFVIII) on protein stability and aggregation. We studied the structural characteristics of the B-domain, and compared FL-rFVIII, pdFVIII and purified rFVIII molecular species with variable B-domain content with regard to their aggregation behaviour upon physical stress. Based on our observations, we built a schematic model of FL-rFVIII and BDD-rFVIII aggregation and suggest a new role for the B-domain and molecular heterogeneity in ensuring the stability of the FVIII molecule.

## Materials and Methods

### FVIII Samples and Chemicals

Human FL-rFVIII (0.23 mg/ml) used was an intermediate material from a production line for a commercial rFVIII product expressed in Chinese hamster ovary (CHO) cells and was provided by Baxalta Innovations GmbH, a Takeda company, Vienna, Austria. Vials of historical lots of commercially available human rFVIII as well as commercially available lyophilised human plasma FVIII product were provided by Baxalta Innovations GmbH, a Takeda company, Vienna, Austria. Chemicals were purchased from Sigma-Aldrich, St. Louis, MO, USA.

### Purification of pdFVIII and rFVIII Molecular Species

Von Willebrand factor (VWF)-free pdFVIII was purified from a commercially available lyophilised plasma FVIII product, which was provided by Baxalta Innovations GmbH, a Takeda company, Vienna, Austria. Multiple vials of the commercially available lyophilised human pdFVIII product were reconstituted and pooled to achieve a homogeneous starting material. FVIII was captured on an anti-FVIII affinity column and further processed using strong cation exchange chromatography. Another purification step on a strong anion exchange resin was performed for buffer exchange and to increase the FVIII concentration.

rFVIII molecular species with different degrees of B-domain truncation were isolated from an intermediate material of a commercially available human FL-rFVIII product expressed in CHO cells, which was provided by Baxalta Innovations GmbH, a Takeda company, Vienna, Austria. A high-resolution anion exchange chromatography step with a flat gradient was used to pre-separate the entities. Pools with enriched sub-species were generated and further purified by preparative size-exclusion chromatography (SEC), followed by concentration and buffer exchange on a strong anion exchange resin.

### Sodium Dodecyl Sulfate–Polyacrylamide Gel Electrophoresis (SDS-PAGE)

SDS-PAGE was carried out using Novex NuPAGE (Thermo Fisher Scientific, Waltham, MA, USA). Samples were mixed with 0.5 M iodoacetamide and incubated for 30 min at 37°C. NuPAGE lithium dodecyl sulfate (LDS) sample buffer and NuPAGE reducing agent were added to the reaction mixture and incubation was continued for 30 min at 37°C. Samples and Precision Plus unstained protein standard (Bio-Rad, Hercules, CA, USA) were loaded onto a 7% Tris-acetate mini gel. Electrophoresis was performed for 90 min at 150 V. Protein bands were visualised with SilverQuest silver staining kit (Thermo Fisher Scientific).

### ***In Silico*** Protein Analysis

BioAnnotator from Vector NTI Advance 11 (Thermo Fisher Scientific, Waltham, MA, USA) was used to calculate the average hydropathicity of the B-domain.

### Hydrogen/Deuterium Exchange Mass Spectrometry

Local amide hydrogen/deuterium exchange (HDX) kinetics were followed after 3, 10 and 30 s; 2, 10 and 60 min; 3 h; and 3 days of incubation. All HDX reactions were performed at 22°C, except for the 3-s reaction (6°C). Human rFVIII containing 70% B-domain (B70-rFVIII) was mixed with deuterated buffer (Tris, pH 6.7, containing CaCl_2_ and NaCl). The reaction was stopped with ice-cold phosphate buffer, pH 2.3, containing 100 mM Tris(2-carboxyethyl)phosphine and 3.3 M urea, and by subsequent snap freezing in liquid nitrogen. Samples were digested using a high-performance liquid chromatography (HPLC) column (ACE, Aberdeen, UK) packed with pepsin-agarose from porcine gastric mucosa (Sigma-Aldrich) and desalted on a C18 pre-column (ACE). Peptic peptides were subjected to liquid chromatography coupled to mass spectrometry (MS) using a HALO C18/1.8 μm column (Advanced Materials Technology, Wilmington, DE, USA). Peptides were eluted by an acetonitrile gradient and analysed on an Orbitrap XL MS (60,000 resolution at m/z 400; Thermo Fisher Scientific). Peptic peptides were identified by three independent liquid chromatography-MS/MS analyses of a non-deuterated sample using the same procedure as for the deuterated samples.

### FVIII Chromogenic Activity

FVIII samples were dialysed against phosphate-buffered saline containing 0.9 mM CaCl_2_ and 0.5 mM MgCl_2_. FVIII activity was measured by chromogenic assay using commercially available reagents (Siemens Healthcare, Erlangen, Germany) on an automated coagulation analyser (BCS XP; Siemens Healthcare). The reference standard was commercially available FL-rFVIII (Baxalta Innovations GmbH, a Takeda company), calibrated against the World Health Organization international standard.

### Preparation of FVIII Aggregates

All FVIII samples were dialysed against phosphate-buffered saline containing 0.9 mM CaCl_2_ and 0.5 mM MgCl_2_. To ensure reproducibility, all experiments were performed at least twice.

#### Temperature-Dependent Aggregation

All FVIII samples with protein concentrations of either 0.122 μM for high-performance size-exclusion chromatography (HPLC-SEC) or 0.61 μM for dynamic light scattering (DLS) were incubated at 25, 30, 35, 40, 45 or 50°C for 20 h in polystyrene microplates (Corning Incorporated – Life Sciences, Tewksbury, MA, USA) covered with plate sealers in a Synergy H4 Hybrid Reader (BioTek, Winooski, VT, USA) with 20 s medium shaking every 10 min. Samples were subsequently frozen at −80°C until analysis.

#### Time-Dependent Aggregation at 45°C

All FVIII samples (0.122 μM) were incubated at 45°C for 24 h in polystyrene microplates (Corning) covered with plate sealers in a plate thermo shaker (Biosan, Riga, Latvia). Samples were withdrawn after various time intervals and immediately frozen at −80°C until HPLC-SEC analysis.

#### Homologous Seeding of FVIII Aggregation

To prepare seeds, FVIII samples (0.122 μM) were incubated for 2, 5, 8 or 18 h at 45°C in polystyrene microplates (Corning) covered with plate sealers in a plate thermo shaker (Biosan). Native FVIII samples (0.122 μM) were mixed 1:1 with the corresponding seeds and time-dependent aggregation at 45°C was initiated. Samples were stored at −80°C until HPLC-SEC analysis.

#### Agitation and Shear Stress-Induced Aggregation

Samples (0.244 μM) were hand agitated for 10 min in a disposable Omnifix syringe (Braun, Melsungen, Germany). Shear stress was induced by injecting the solution through ‘winged infusion sets with needle protection’ (23G × ¾″; L = 35 cm, V = 0.25 ml) from Terumo Europe, Leuven, Belgium, after which all samples were stored at −80°C until flow cytometry-based particle analysis.

### DLS

DLS was performed using a Malvern NanoZetasizer ZSP (Malvern Instruments, Malvern, UK). Samples (0.244 μM) were centrifuged (Centrifuge 5415C; Eppendorf, Vienna, Austria) at 10,000 rpm for 5 min and filled into a ZEN0040 disposable microcuvette. Operation temperature was set at 25°C with an equilibration time of 2 min. The angle was set to 173° backscatter to determine the hydrodynamic diameter of a protein and thus, the effective size of proteins. A minimum of three runs per sample were measured to obtain an average result.

### HPLC-SEC

HPLC-SEC was performed using a 7.8 × 300 mm TSKgel G4000SWxl column (Tosoh Bioscience, Tokyo, Japan) and a 6 × 40 mm TSK guard column (Tosoh Bioscience) coupled to an HPLC 1260 infinity system (Agilent Technologies, Santa Clara, CA, USA). SEC was carried out under isocratic conditions at a flow rate of 0.3 ml/min using a buffer consisting of 50 mM Tris-HCl, 5 mM CaCl_2_, 400 mM NaCl and 0.05% NaN_3_, pH 7.0. A sample volume of 100 μl (0.122 μM protein concentration) was mixed with 3 μl Thioflavin T (ThT; 1 mM) and subsequently loaded onto the column. To monitor the elution of the protein with fluorescence detection, the excitation and emission wavelengths were set to 280 nm and zero order, respectively. ThT fluorescence was monitored with 440-nm excitation and zero-order emission. Peaks eluting with the void volume (retention time 18.0–21.2 min), with retention times of 21.2–27.0 min and 27.0–43.0 min, were designated as soluble protein aggregates, oligomers and monomers, respectively. The amount of each was calculated as a percentage of the total area of all peaks in the chromatogram. ThT binding was calculated as the ratio between ThT and intrinsic protein fluorescence signals. The protein-based gel filtration standard (Bio-Rad) was analysed in between samples to monitor optimal column performance. All samples were analysed in random order.

### Curve Fitting and Statistical Analysis

Curve fitting was computed by GraphPad Prism 6 (GraphPad Software, La Jolla, CA, USA). Kinetic rate constants for oligomerisation (*k*_oligo_ [h^−1^]) were derived by fitting data to the one-phase association model according to: y = y_0_ + (plateau-y_0_)*(1-exp[−*k*_oligo_*x]); y = oligomer amount (%); x = time (h); y_0_ = y value when x is zero; plateau = y value at infinite times. Aggregation rates (*k*_agg_ [h^−1^]) were derived by fitting data to the Boltzmann sigmoidal model according to: y = y_min_ + (y_max_-y_min_)/(1 + exp[(x_1/2_-x)/(1/*k*_agg_)]); y = aggregate amount (%); y_min_ = y during lag phase; y_max_ = y after aggregation ended; x = time (h); x_1/2_ = time at half-maximum y (33).

The statistical differences in Figure 6 were computed by GraphPad Prism 6 using unpaired t-test.

### Flow Cytometry-Based Particle Analysis

A flow cytometry-based particle analysis method was used to detect sub-visible particles 0.75–70 μm in size, as previously described (34). A combination of size calibration beads (Fluoresbrite® YG Carboxylate Size Range beads; Polysciences Inc., Warrington, PA, USA), counting beads (CountBright™ Absolute Counting Beads; Invitrogen Corporation, Carlsbad, CA, USA) and fluorescent probes was used to characterise sub-visible particles. To distinguish protein and protein-containing particles from non-protein sub-visible particles, samples were stained with the fluorescent dye 4,4′-Dianilino-1,1′-binaphthyl-5,5′-disulfonic acid dipotassium salt (Bis-ANS).

## Results

### Similar Heterogeneity of pdFVIII and FL-rFVIII

Figure 1a provides a schematic overview of the multi-domain structure of FVIII. Brackets indicate molecular species resulting from complex post-translational processing within the B-domain. rFVIII molecular species containing 100% (B100-), 70% (B70-), 20% (B20-) or 0% B-domain were the main rFVIII species found in FL-rFVIII, with HC migration levels at 180, 150, 110 and 90 kDa, respectively (Fig. 1b). Percentages of B-domain content (100%, 70% or 20%) in rFVIII species nomenclature were calculated on the basis of the apparent molecular mass of the B-domain of the respective rFVIII HC derived from the SDS-PAGE gel migration levels. The B-domain of the B100-HC (180 kDa, lane 1) was calculated to have 90 kDa and contain 100% of total B-domain; that of the B70-HC (150 kDa, lane 3) was 60 kDa and ~70% of total B-domain, and that of the B20-HC (110 kDa, lane 4) was 20 kDa and ~20% of total B-domain.

pdFVIII isolated from pooled human plasma was highly purified, whereby VWF was depleted to 7.5 μg VWF/mg FVIII. pdFVIII and CHO-derived FL-rFVIII showed almost identical heterogenic protein profiles on the silver-stained SDS-PAGE gel (Fig. 1b, lanes 1–2). Both displayed the most intense band at ~180 kDa, indicating glycosylated B100-HC species, and several truncated HC/B-domain species with lower molecular weight migrating at comparable levels. The presence of the main molecular FVIII species in FL-rFVIII and pdFVIII was confirmed in SEC profiles (Fig. S1). In addition, the heterogeneity in FL-rFVIII was consistent for lots produced between 2005 and 2015 (Fig. 1c).

Specific FVIII activity of FL-rFVIII and pdFVIII was 4380 ± 868 and 4457 ± 493 IU/mg using chromogenic assay.

### Purification and Characterisation of rFVIII Molecular Species

BDD-rFVIII, B20-rFVIII and B70-rFVIII were isolated to 95%, 94% and 92% purity, respectively, based on C4 HPLC analysis (data not shown). The HC of B70-rFVIII, B20-rFVIII and BDD-rFVIII exhibited apparent molecular weights of 150, 110 and 90 kDa on the SDS-PAGE gel, due to varying amounts of B-domain (Fig. 1b, lanes 3–5). An apparent molecular weight band of 75 kDa was observed for the LC of each species. The integrity of the purified rFVIII species was confirmed by HPLC-SEC (Fig. S1). B100-FVIII could not be purified to sufficient quantity and was not used in this study.

Specific FVIII activity was 5088 ± 403, 5202 ± 317 and 6261 ± 263 IU/mg for BDD-rFVIII, B20-rFVIII and B70-rFVIII, as measured by chromogenic assay.

### B-Domain Structural Characteristics

The B-domain has an amino acid sequence with low overall hydrophobicity. The average hydropathicity calculated according to the Kyte-Doolittle method (35) was −0.751 for the total sequence. Clusters of low hydrophobicity were evenly distributed. B-domain sequences in B100-, B70- and B20-rFVIII exhibited similar average hydropathicity values of −0.779, −0.741 and − 0.896, respectively. Low hydrophobicity characterises natively unfolded proteins (36).

B70-rFVIII was subjected to HDX-MS; the kinetics of 120 peptides from the total protein sequence, including nine peptides from the B-domain sequence, were measured (Table S1). All peptides from sequences belonging to the B-domain demonstrated rapid kinetics of deuterium incorporation. Even at the shortest incubation time (3 s), all peptides incorporated the same amount of deuterium as the corresponding fully deuterated sample after 3 days.

The HDX-MS data together with the amino acid sequence characteristics indicate that the B-domain of B70-rFVIII is intrinsically disordered and flexible. Based on the *in silico* analysis, similar solvent exposure and flexibility is expected for the total B-domain and several B-domain truncations present in FL-rFVIII and pdFVIII.

### Aggregation Behaviour of FL-rFVIII and rFVIII Molecular Species at Elevated Temperatures

**Fig. 2:**
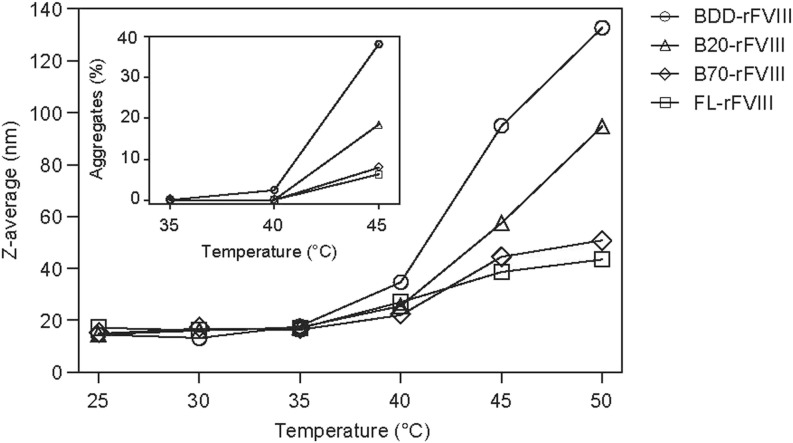
**Aggregation of rFVIII at elevated temperature.** Thermally induced aggregates of FL-rFVIII (□), B70-rFVIII (◊), B20-rFVIII (Δ) and BDD-rFVIII (○) were analysed by dynamic light scattering. The inset depicts the corresponding samples analysed by high-performance size-exclusion chromatography. B20-/B70-rFVIII, human recombinant factor VIII containing 20%/70% B-domain, respectively; BDD-rFVIII, human B-domain-deleted recombinant factor VIII; FL-rFVIII, human full-length cDNA-based recombinant factor VIII; rFVIII, recombinant factor VIII.

FL-rFVIII, B70-rFVIII, B20-rFVIII and BDD-rFVIII were exposed to temperatures of 25–50°C and analysed by DLS and HPLC-SEC (Fig. 2). Starting at 40°C, an increase in Z-average, describing the intensity weighted mean hydrodynamic diameter of protein aggregates, was observed for all items, with clear differences between rFVIII samples. The mean aggregate size increased with decreasing B-domain content (aggregation propensity order BDD-rFVIII > B20-rFVIII > B70-rFVIII > FL-rFVIII). FL-rFVIII, being a heterogenic mixture, exhibited the lowest propensity to aggregate. The fold increase of the aggregate average size was 9.2 for BDD-rFVIII and 6.4 for B20-rFVIII, but only 3.4 for B70-rFVIII and 2.5 for FL-rFVIII. The same trend in aggregation was shown by HPLC-SEC analysis (Fig. 2 inset). Furthermore, SDS-PAGE showed that FL-rFVIII aggregates, separated from FL-rFVIII monomers by HPLC-SEC, contained each rFVIII molecular species in a similar ratio to native FL-rFVIII (Fig. S2).

### FVIII Aggregation Pathways Depend on Molecular Heterogeneity and B-Domain Content

**Fig. 3:**
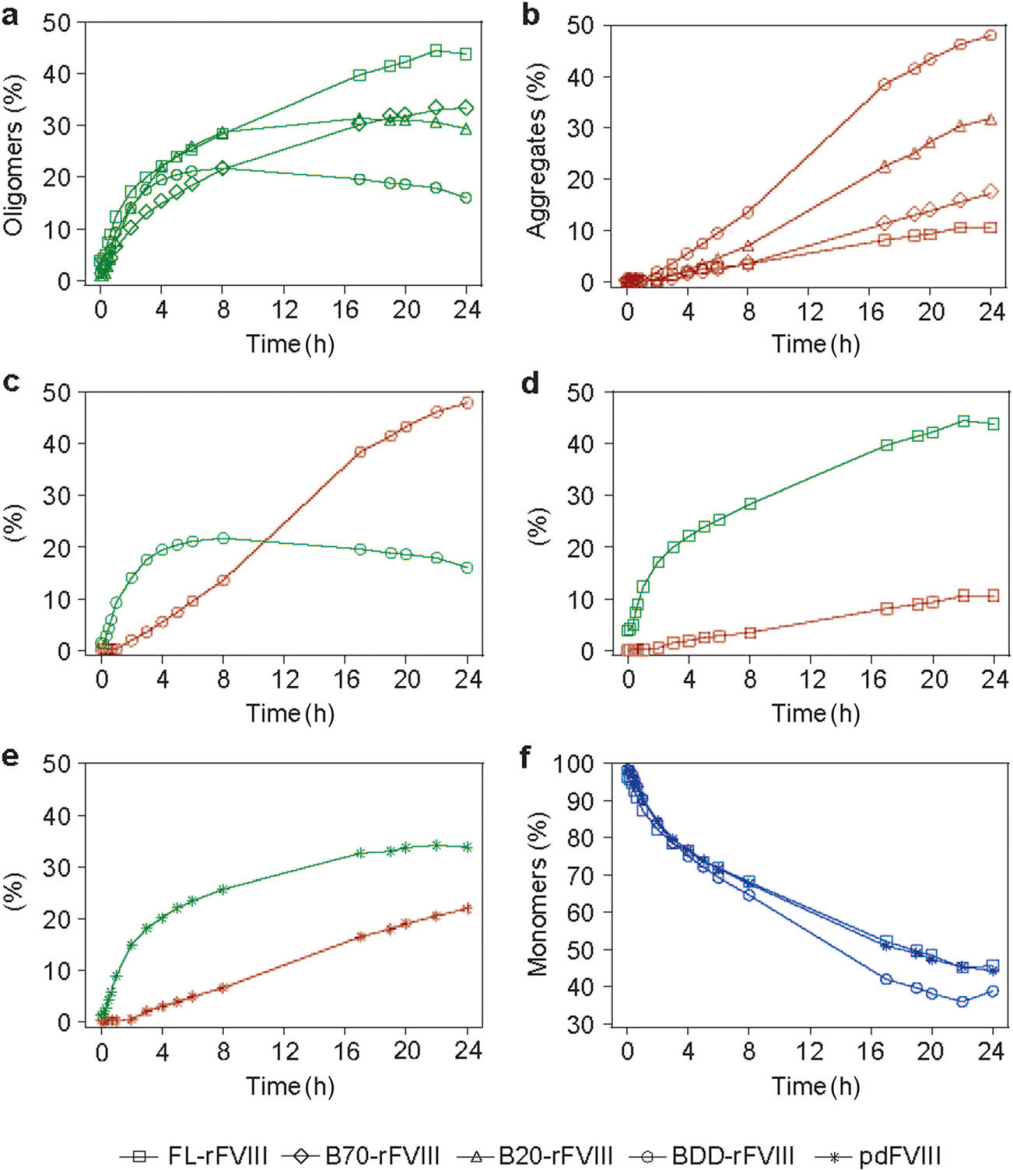
**Pathways of rFVIII oligomer and aggregate formation.** FL-rFVIII (□, **d**), B70-rFVIII (◊), B20-rFVIII (Δ), BDD-rFVIII (○, **c**) and pdFVIII (*, **e**) were incubated at 45°C for 24 h. The amount of oligomers (green, **a**), aggregates (red, **b**) and monomers (blue, **f**) was continuously analysed by high-performance size-exclusion chromatography and plotted against time of incubation. B20-/B70-rFVIII, human recombinant factor VIII containing 20%/70% B-domain, respectively; BDD-rFVIII, human B-domain-deleted recombinant factor VIII; FL-rFVIII, human full-length cDNA-based recombinant factor VIII; pdFVIII, human plasma-derived factor VIII; rFVIII, recombinant factor VIII.

Detailed time-dependent aggregation analysis was performed at 45°C, the temperature at which conformational changes in the FVIII molecule are initiated (37, 38). Aggregation kinetics were followed by HPLC-SEC and showed clear differences in aggregation and oligomerisation pathways between rFVIII molecular species and FL-rFVIII (Fig. 3). Based on the exclusion limit of the analysis column, oligomers were 10–50 nm and aggregates 50–100 nm in size. Oligomerisation of all samples followed a one-phase association curve, but with different kinetics (Fig. 3a). The rate constants for oligomerisation (*k*_oligo_) of FL-rFVIII, B70-rFVIII, B20-rFVIII and BDD-rFVIII were 0.13 ± 0.02, 0.12 ± 0.05, 0.35 ± 0.24 and 0.70 ± 0.24 h^−1^, respectively. Aggregation curves had sigmoidal shapes with varying aggregation rates (*k*_agg_), depending on B-domain content (Fig. 3b). With no B-domain, aggregates formed more rapidly and excessively: *k*_agg_(FL-rFVIII) = 0.11 ± 0.00 h^−1^, *k*_agg_(B70-rFVIII) = 0.18 ± 0.02 h^−1^, *k*_agg_(B20-rFVIII) = 0.21 ± 0.07 h^−1^ and *k*_agg_(BDD-rFVIII) = 0.21 ± 0.08 h^−1^. Aggregation pathways of FL-rFVIII and BDD-rFVIII diverged the most (Fig. 3c, d). pdFVIII (Fig. 3e) followed a similar pathway to FL-rFVIII, with rate constants of *k*_oligo_(pdFVIII) = 0.24 ± 0.04 h^−1^ and *k*_agg_(pdFVIII) = 0.15 ± 0.03 h^−1^*.* Consistent with these oligomerisation and aggregation rates, the loss of monomers in BDD-rFVIII was faster than in FL-rFVIII and pdFVIII (Fig. 3f).

### Diverging Aggregation Pathways Triggered by Structurally Different Aggregates

**Fig. 4:**
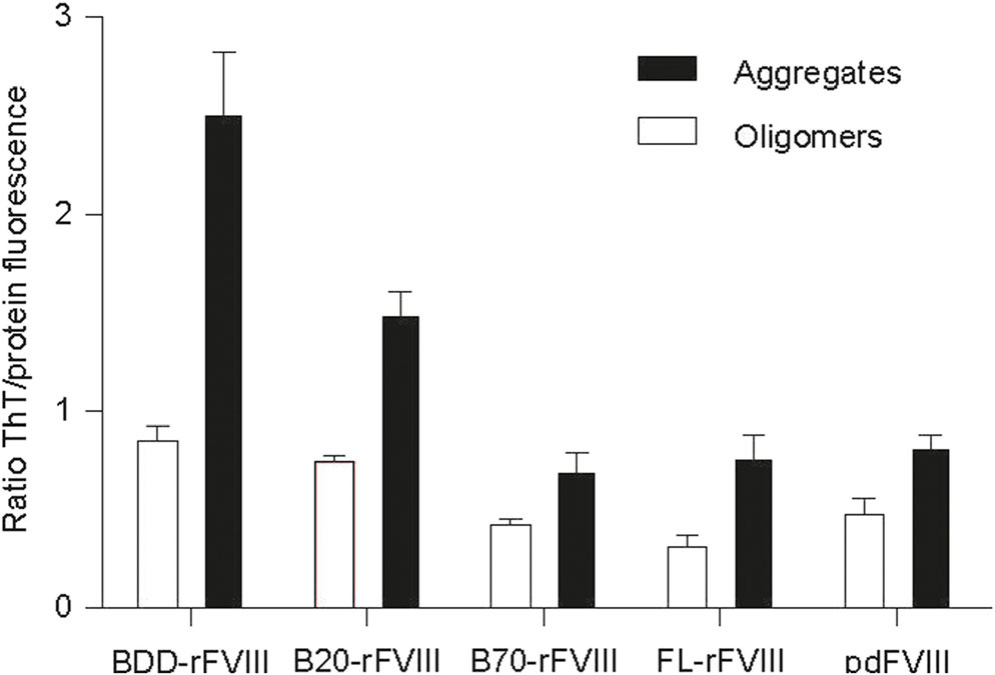
**Binding of the fluorescent dye ThT to oligomers and aggregates of FVIII.** The binding capacity of ThT to protein oligomers and aggregates is expressed as the ratio of the fluorescent signals at 440- and 280-nm excitation after 24 h of incubation at 45°C. *n* = 2–4, error bars indicate SD values. B20-/B70-rFVIII, human recombinant factor VIII containing 20%/70% B-domain, respectively; BDD-rFVIII, human B-domain-deleted recombinant factor VIII; FL-rFVIII, human full-length cDNA-based recombinant factor VIII; FVIII, factor VIII; pdFVIII, human plasma-derived factor VIII; ThT, Thioflavin T.

ThT is a commonly used fluorescent dye that displays enhanced fluorescence upon binding to cross-β sheet-rich structures (39). The binding capacity of ThT to aggregated FVIII protein structures was expressed as the ratio of ThT to intrinsic protein fluorescence (Fig. 4). ThT binding with oligomers of BDD-rFVIII was higher than for B-domain-containing species, FL-rFVIII and pdFVIII. Aggregates of BDD-rFVIII exhibited ThT binding ability three-fold greater than FL-rFVIII and pdFVIII. Furthermore, ThT fluorescence with B20-rFVIII aggregates was greater than with B70-rFVIII aggregates; the fluorescence with FL-rFVIII or pdFVIII oligomers and aggregates was similar (Fig. 4). ThT binding to monomers was not detected in any FVIII sample.

### Homologous Seeding of FVIII Aggregation

**Fig. 5:**
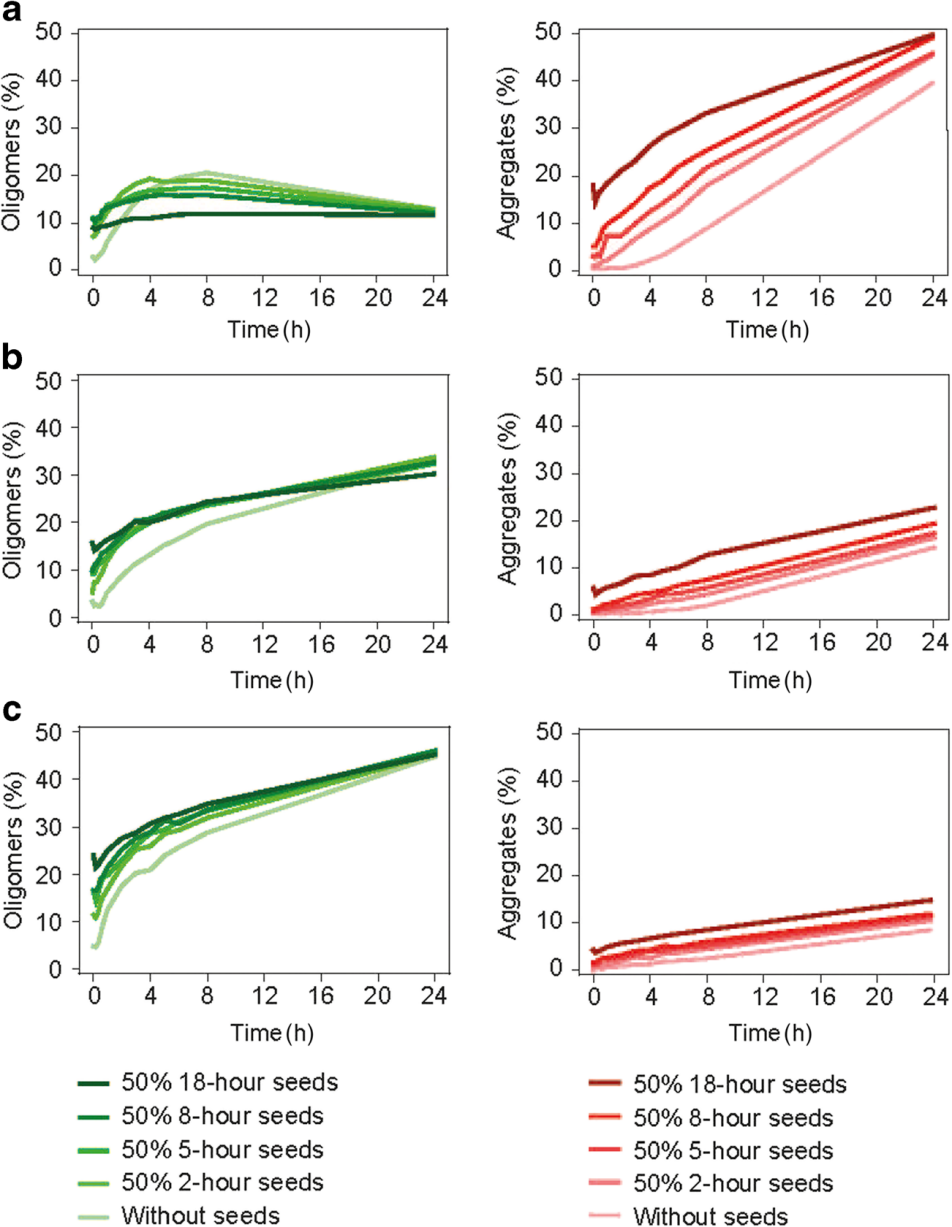
**Homologous seeding of rFVIII aggregation.** Seeds were prepared by incubation of BDD-rFVIII, B70-rFVIII and FL-rFVIII for 2, 5, 8 or 18 h at 45°C. BDD-rFVIII (**a**), B70-rFVIII (**b**) and FL-rFVIII (**c**) samples were mixed 1:1 with respective pre-formed seeds and incubated at 45°C for 24 h. The amount of oligomers (green) and aggregates (red) was continuously analysed by high-performance size-exclusion chromatography and plotted against incubation time. B70-rFVIII, human recombinant factor VIII containing 70% B-domain; BDD-rFVIII, human B-domain-deleted recombinant factor VIII; FL-rFVIII, human full-length cDNA-based recombinant factor VIII; rFVIII, recombinant factor VIII.

Cross-β sheet-containing aggregates serve as seeds, which nucleate further protein aggregation upon stress (40,41). Investigation of the ability of aggregated BDD-rFVIII, B70-rFVIII and FL-rFVIII to seed the aggregation process showed that homologous seeding of B70-rFVIII and heterogeneous FL-rFVIII (Fig. 5b, c) did not alter oligomerisation or aggregation behaviour. BDD-rFVIII exhibited a different mechanism: after initial rapid oligomerisation, the rate flattened with addition of homologous seeds until reaching a saturation concentration of 10%. The lag phase of BDD-rFVIII aggregation decreased according to the type of seeds added. After adding the seeds, generated by incubation for 8 or 18 h at 45°C, the lag phase of BDD-rFVIII aggregation curves vanished completely (Fig. 5a). A reduction in lag phase is typical of nucleation-dependent polymerisation and has been previously described for several proteins including insulin (42,43).

### Formation of Sub-visible FVIII Particles under Agitation and Shear Stress

**Fig. 6:**
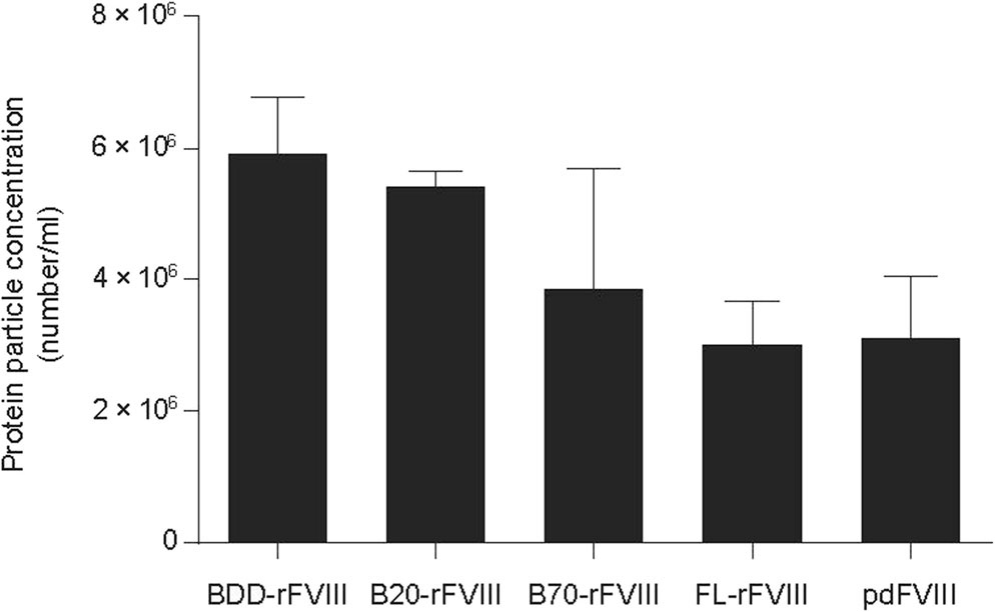
**Formation of protein-containing sub-visible particles.** FVIII samples (0.244 μM) were exposed to agitation and shear stress and subjected to flow cytometry-based particle analysis. Statistical differences were shown by using unpaired t-test. Protein particle concentrations were significantly different between BDD-rFVIII and FL-rFVIII (*P* = 0.0002), BDD-rFVIII and pdFVIII (*P* = 0.0010), B20-rFVIII and FL-rFVIII (*P* < 0.0001) and B20-rFVIII and pdFVIII (*P* < 0.0001). *n* = 4–6, error bars indicate SD values. B20-/B70-rFVIII, human recombinant factor VIII containing 20%/70% B-domain, respectively; BDD-rFVIII, human B-domain-deleted recombinant factor VIII; FL-rFVIII, human full-length cDNA-based recombinant factor VIII; FVIII, factor VIII; pdFVIII, human plasma-derived factor VIII.

FL-rFVIII and pdFVIII showed similar concentrations of protein-containing particles: 2.4–4.2 × 10^6^ counts/ml (mean values 3.1 × 10^6^ counts/ml and 3.2 × 10^6^ counts/ml, respectively). BDD-rFVIII had a significantly higher concentration (mean value 6.0 × 10^6^ counts/ml; range 4.9–6.9 × 10^6^ counts/ml). The sub-visible protein-containing particle concentration of B-domain truncated molecular species depended on the B-domain content of the test species and was higher for B20-rFVIII (mean value 5.5 × 10^6^ counts/ml) than for B70-rFVIII (mean value 3.9 × 10^6^ counts/ml) (Fig. 6). Non-stressed samples resulted in sub-visible protein particle concentrations of 0.4–4.6 × 10^4^ counts/ml.

## Discussion

In this first study investigating the heterogeneity, stability and aggregation behaviour of human FL-rFVIII compared with highly purified human pdFVIII, or purified rFVIII molecular species containing variable amounts of B-domain, we explored the structural characteristics of the B-domain and its role in stabilising the FVIII molecule.

Our data indicate that FL-rFVIII produced in CHO cells displays a natural heterogeneity similar to pdFVIII and has all the molecular B-domain species also present in pdFVIII. rFVIII molecular species containing 100% (B100-), 70% (B70-), 20% (B20-) or 0% B-domain were the main rFVIII species found in FL-rFVIII and pdFVIII in the present study. Jankowski *et al*. previously described the terminating amino acid positions of the B-domain truncations of the main rFVIII species present in FL-rFVIII as Arg^1313^, Arg^1115^, Ser^817^ and Ser^740^, which likely correspond to the terminating amino acids of B100-, B70-, B20- and BDD-rFVIII HCs presented in this study. The B-domain part spanning from Arg^1313^ to Arg^1648^ is most likely completely removed after cleavage as it was not detected among the secreted FVIII HC species (5).

Interestingly, FL-rFVIII produced in a baby hamster kidney cell line exhibits a slightly different protein profile regarding band intensity and migration level than CHO cell-derived FL-rFVIII and pdFVIII (5). In general, FVIII’s heterogeneity, even with minor differences in the HC/B-domain truncation length, is species independent. This was true for human pdFVIII and FL-rFVIII in this and previous work (5) and also for porcine pdFVIII (44). In contrast, the purified BDD-rFVIII species used in this study, and marketed BDD-rFVIII products, exhibit a monogenic protein pattern and thus large differences from heterogenic pdFVIII (6–8).

FL-rFVIII and pdFVIII demonstrated the lowest propensity to form aggregates. The aggregation tendency increased with decreasing B-domain content of the rFVIII molecular species. Detailed time-dependent analysis of oligomerisation and aggregation under thermal stress revealed diverging pathways for different FVIII samples. The slow oligomerisation of thermally stressed FL-rFVIII and pdFVIII nearly inhibited aggregation. The kinetics of oligomer and aggregate formation were more rapid with BDD-rFVIII. ThT-positive cross-β sheet-rich structures were detected in thermally induced BDD-rFVIII oligomers and aggregates, but less so or not at all in B-domain-containing FVIII. It is likely that cross-β sheets in BDD-rFVIII oligomers trigger extensive aggregation and facilitate homologous seeding of BDD-rFVIII aggregation, but not in the other FVIII samples.

**Fig. 7:**
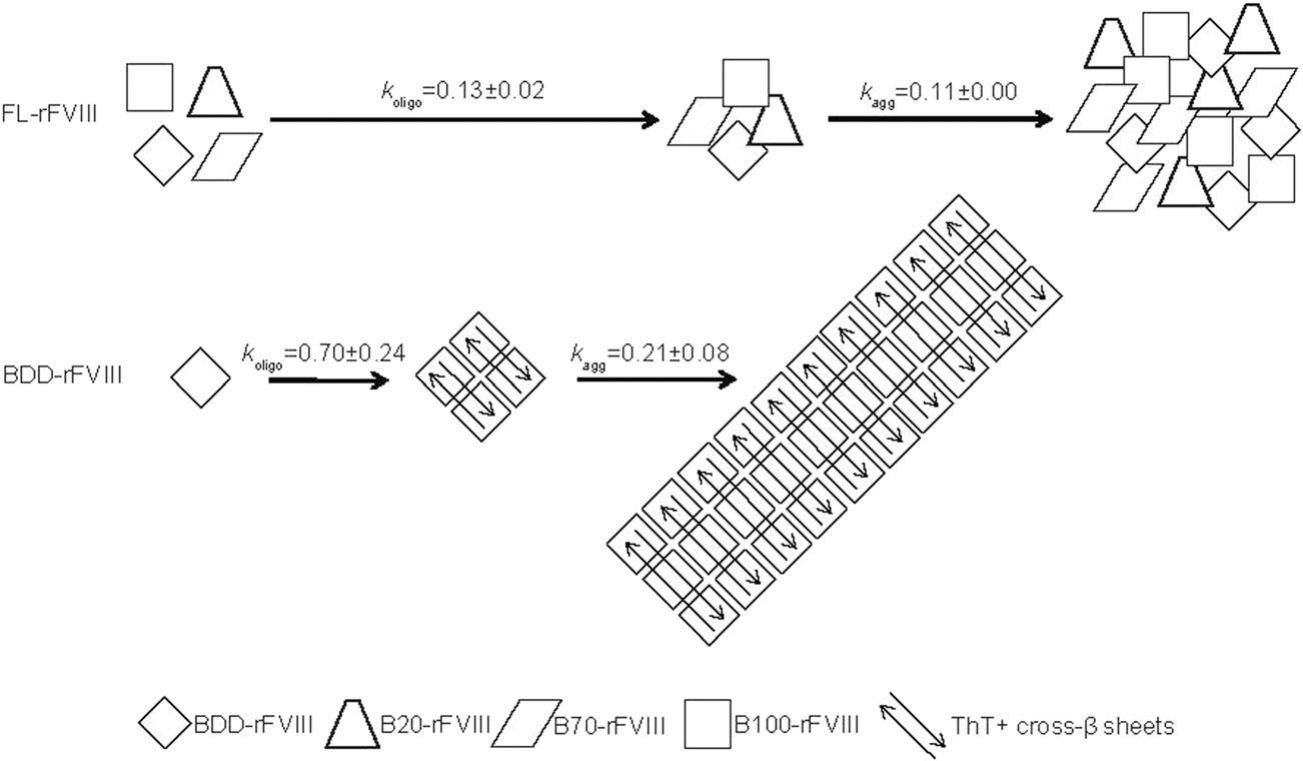
**Schematic model of oligomer and aggregate formation after exposure of rFVIII to thermal stress.** FL-rFVIII is depicted as a heterogeneous mixture of rFVIII species, but does not reflect the actual ratio of species. The length of arrows indicates the differences in oligomerisation and aggregation rates of FL-rFVIII and BDD-rFVIII. B20-/B70-/B100-rFVIII, human recombinant factor VIII containing 20%/70%/100% B-domain, respectively; BDD-rFVIII, human B-domain-deleted recombinant factor VIII; FL-rFVIII, human full-length cDNA-based recombinant factor VIII; rFVIII, recombinant factor VIII; ThT, Thioflavin T.

Based on these observations, we built a schematic model describing the diverging pathways of BDD-rFVIII and FL-rFVIII oligomer and aggregate formation (Fig. 7). While the starting material in FL-rFVIII is a heterogenic mixture of all rFVIII species, BDD-rFVIII exists only as one species. Arrow lengths in the model indicate oligomerisation and aggregation rates, both of which are much faster for BDD-rFVIII than for FL-rFVIII. While BDD-rFVIII forms ordered, large cross-β sheet-rich aggregates, FL-rFVIII aggregates lack this repetitive nature and are smaller in size. The assembly of soluble proteins into ordered cross-β sheet-containing structures is seen in many human neurodegenerative diseases, such as Alzheimer’s disease and Parkinson’s disease, and in spongiform encephalopathies (45). Development of such disorders has been associated with the seeding ability of respective accumulated protein aggregates (41).

Supported by a previous study in which the B-domain had little to no effect on the overall FVIII secondary structure in solution as measured by far UV circular dichroism spectroscopy (32), the present study demonstrates that the B-domain is solvent exposed, disordered and flexible. We thus propose that the B-domain has an aggregation-protective function for FVIII, similar to that in other proteins with significantly disordered segments, such as α-synuclein. The natively unfolded C-terminal region of α-synuclein was shown to be essential in stabilising the protein. Aggregation of α-synuclein was clearly dependent on the length of the C-terminal region and decreased with increasing content of the disordered region (46–48).

Heterogeneity, as shown in FL-rFVIII and pdFVIII, causes lower sequence similarity between FVIII species. Sequence diversity in proteins has already been demonstrated in previous studies as being essential in reducing aggregation susceptibility and seeding processes. Wright and co-workers’ findings on the multi-domain protein titin showed that the co-aggregation efficiency among different domains decreases markedly with decreasing sequence identity. Furthermore, they claimed that maintaining a low sequence identity between proteins is an important evolutionary characteristic that strongly inhibits aggregation in the crowded environment of a living system (49). Similarly, the efficiency of seeding in the fibril formation of lysozymes was shown to strongly depend on the similarity of their sequences (50).

Using agitation and shear stress to mimic mishandling of FVIII therapeutic products, and facilitation of the interaction of proteins with silicone oil, which is often present on the interior surfaces of syringes (34,51–53), showed that sub-visible protein-containing particle formation is clearly dependent on B-domain content. Particle concentrations in FL-rFVIII and pdFVIII reached similar levels, whereas significantly more particles were detected in BDD-rFVIII.

Previously published work demonstrated that glycosylation significantly influences the stability of FL-rFVIII, as shown by reduced aggregation resistance of de-glycosylated FL-rFVIII (54). Given that ~80% of the *N*-glycosylation sites are distributed within the B-domain (23), deletion of this domain is likely to render the protein more susceptible to aggregation. Manufacturing and formulation specifications further influence aggregation of FVIII and protein therapeutics (55). Large differences in quality attributes, such as aggregate and sub-visible particle concentrations, were observed for marketed rFVIII products that differ mainly in B-domain structure and manufacturing processes (56).

Protein aggregates not only influence protein drugs’ stability and shelf-life, but also increase their immunogenicity (55). The repetitive nature of protein aggregates can be detected by pattern recognition receptors or cross-link antigen receptors on immune cells. Anti-drug antibodies may have a neutralising effect on the protein, which may affect its potency or pharmacokinetics, and especially in therapeutics related to an endogenous protein, pose patient safety risks (12). Inhibitory antibodies are formed in approximately one-fifth of patients with haemophilia A treated with FVIII (57,58). *In vivo* studies in haemophilia A mice showed that protein aggregates modulate FVIII immunogenicity differently, depending on the nature of the aggregates and how they were formed (59). However, to date no data are available on how protein aggregates in FVIII products modulate their immunogenicity in humans or on the immunogenic properties of the oligomers and aggregates characterised in this study.

## Conclusion

In summary, our data demonstrate similar levels of protein heterogeneity in FL-rFVIII and pdFVIII, and suggest a beneficial effect thereof in reducing the protein aggregation susceptibility upon exposure to physical stress. The B-domain was shown to be involved in ensuring the stability of the FVIII molecule by modulating the protein aggregation pathway. These findings should be considered in the design of future FVIII therapeutics to improve their stability, shelf-life and, most importantly, their safety.

## Electronic supplementary material

ESM 1 (DOCX 179 kb)

## Abbreviations

B20-rFVIII: 20% B-domain recombinant factor VIII
B70-rFVIII: 70% B-domain recombinant factor VIII
B100-rFVIII: 100% B-domain recombinant factor VIII
BDD-rFVIII: B-domain-deleted recombinant factor VIII
CHO: Chinese hamster ovary
DLS: Dynamic light scattering
FL-rFVIII: Full-length recombinant factor VIII
FVIII: Factor VIII
HC: Heavy chain
HDX: Hydrogen/deuterium exchange
HPLC: High-performance liquid chromatography
LC: Light chain
MS: Mass spectrometry
pdFVIII: Plasma-derived factor VIII
rFVIII: Recombinant factor VIII
SDS-PAGE: Sodium dodecyl sulfate–polyacrylamide gel electrophoresis
SEC: Size-exclusion chromatography
ThT: Thioflavin T
VWF: Von Willebrand factor
