# Supplementary material for: How Full-Length FVIII Benefits from Its Heterogeneity – Insights into the Role of the B-Domain
Source: Pharm Res. 2019 Apr 1;36(5):77. doi: 10.1007/s11095-019-2599-2 (PMC6443606; doi:10.1007/s11095-019-2599-2)
Supplement: Supplementary file 1 — (DOCX 179 kb) [file 11095_2019_2599_MOESM1_ESM.docx]

**Supplementary figures and table**


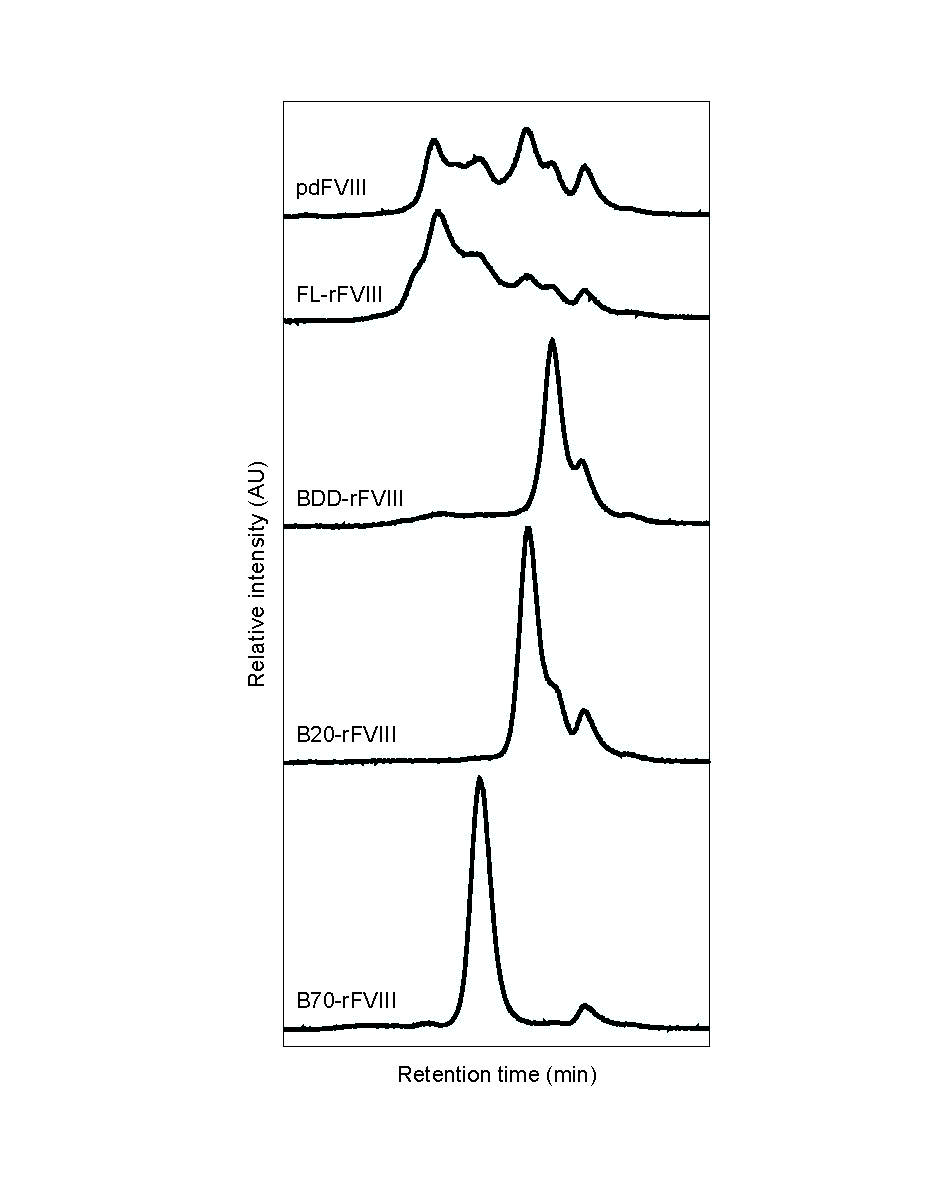


**Fig. S1 Size-exclusion chromatographic profiles of highly purified pdFVIII, FL‑rFVIII and purified rFVIII molecular species at equimolar concentrations.** B20/B70‑rFVIII, human recombinant factor VIII containing 20%/70% B-domain; BDD‑rFVIII, human B-domain-deleted recombinant factor VIII; FL‑rFVIII, human full-length cDNA-based recombinant factor VIII; pdFVIII, human plasma-derived factor VIII.


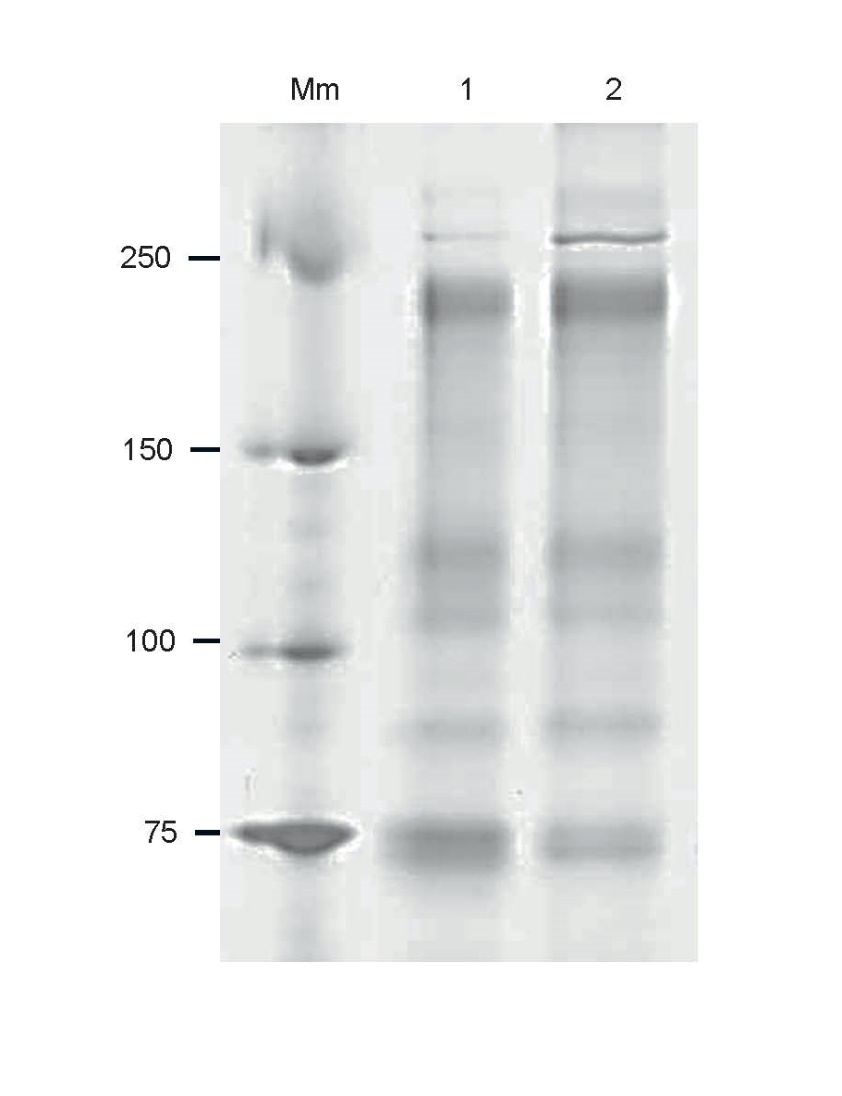


**Fig. S2** **Composition of FL‑rFVIII aggregates.** Silver-stained SDS-PAGE gel of native FL‑rFVIII (1) and purified aggregates of FL‑rFVIII (2). FL‑rFVIII, human full-length cDNA-based recombinant factor VIII; Mm, Precision Plus unstained protein standard (Bio-Rad).

Table S1 Hydrogen/Deuterium Exchange Mass Spectrometry Kinetics of B70‑rFVIII^a^

|  | % of deuterium incorporation after | | |
| --- | --- | --- | --- |
| Peptide sequence | **3 seconds** | **2 minutes** | **3 hours** |
| Heavy chain peptides: |  |  |  |
| 1-ATRRYYLGAVE-11 | 19 | 37 | 60 |
| 1-ATRRYYLGAVELSWDYMQSDL-21 | 33 | 54 | 83 |
| 1-ATRRYYLGAVELSWDYMQSDLGELPVD-27 | 38 | 58 | 74 |
| 45-VYKKTLF-51 | 26 | 91 | 104 |
| 52-VEFTDHLFN-60 | 6 | 47 | 96 |
| 52-VEFTDHLFNIAKPRPPWMGLLGPTIQ-77 | 26 | 63 | 87 |
| 55-TDHLFNIAKPRPPWMGLLGPTIQ-77 | 29 | 67 | 88 |
| 59-FNIAKPRPPWMGLLGPTIQ-77 | 33 | 69 | 88 |
| 59-FNIAKPRPPWMGLLGPTIQA-78 | 31 | 67 | 87 |
| 83-TVVITLKNMASHPVSL-98 | 11 | 23 | 77 |
| 86-ITLKNMASHPVSL-98 | 11 | 30 | 69 |
| 106-WKASEGAE-113 | 25 | 70 | 84 |
| 114-YDDQTSQREKEDDKVFPGGSHT-135 | 33 | 63 | 82 |
| 114-YDDQTSQREKEDDKVFPGGSHTYV-137 | 27 | 47 | 74 |
| 159-LSHVDLVKDLNSGL-172 | 50 | 54 | 93 |
| 165-VKDLNSGLIGAL-176 | 10 | 11 | 10 |
| 185-AKEKTQTLHKFILL-198 | 46 | 65 | 81 |
| 199-FAVFDEGKSWHSETKNSL-216 | 56 | 67 | 81 |
| 203-DEGKSWHSETKNSL-216 | 81 | 90 | 98 |
| 217-MQDRDAASARAWPKM-231 | 81 | 88 | 94 |
| 226-RAWPKMHTVNG-236 | 62 | 72 | 83 |
| 249-HRKSVYWHVIGMGTTPEVHSIF-270 | 19 | 30 | 75 |
| 260-MGTTPEVHSIF-270 | 16 | 32 | 72 |
| 277-LVRNHRQASL-286 | 42 | 56 | 81 |
| 300-LMDLGQF-306 | 8 | 33 | 70 |
| 301-MDLGQFL-307 | 6 | 24 | 47 |
| 309-FCHISSHQHDGMEA-322 | 19 | 44 | 70 |
| 323-YVKVDSCPEEPQL-335 | 84 | 88 | 93 |
| 356-DVVRFDDDNSPSF-368 | 93 | 101 | 100 |
| 369-IQIRSVAKKHPKTWVHY-385 | 60 | 87 | 91 |
| 369-IQIRSVAKKHPKTWVHYIAA-388 | 60 | 84 | 88 |
| 369-IQIRSVAKKHPKTWVHYIAAEEED-392 | 48 | 81 | 90 |
| 393-WDYAPLVLAPDDRS-406 | 65 | 73 | 84 |
| 431-YTDETFKTREAIQHESGILGPLL-453 | 47 | 76 | 90 |
| 436-FKTREAIQHESGILGPLL-453 | 50 | 79 | 86 |
| 441-AIQHESGILGPLL-453 | 25 | 47 | 62 |
| 454-YGEVGDTLL-462 | 3 | 4 | 25 |
| 462-LIIFKNQASRPYNIYPHGITDVRPLY-487 | 31 | 73 | 83 |
| 466-KNQASRPYNIYPHGITD-482 | 22 | 68 | 88 |
| 501-FPILPGEIFKYKWTVT-516 | 1 | 12 | 60 |
| 540-ERDLASGLIGPLL-552 | 30 | 37 | 50 |
| 553-ICYKESVDQRGNQIMSDKRNVIL-575 | 58 | 71 | 79 |
| 566-IMSDKRNVIL-575 | 53 | 85 | 88 |
| 576-FSVFDENRSWYLTEN-590 | 8 | 14 | 20 |
| 580-DENRSWYLTEN-590 | 29 | 60 | 74 |
| 590-NIQRFLPNPAGVQ-602 | 40 | 59 | 81 |
| 590-NIQRFLPNPAGVQLEDPEF-608 | 47 | 70 | 86 |
| 591-IQRFLPNPAGVQ-602 | 53 | 75 | 84 |
| 609-QASNIMHSING-619 | 16 | 40 | 73 |
| 638-YILSIGAQTDF-648 | 22 | 30 | 69 |
| 653-FSGYTFKHKMVYEDT-667 | 8 | 17 | 54 |
| 680-MSMENPGL-687 | 7 | 30 | 81 |
| 680-MSMENPGLWILGCHNSD-696 | 9 | 22 | 55 |
| 706-LKVSSCDKNTGD-717 | 73 | 86 | 90 |
| 731-LSKNNAIEPRS-741 | 100 | 98 | 101 |
| 756-FNATTI-761 | 97 | 102 | 98 |
| 794-LRQSPTPHGLSLSDL-808 | 76 | 85 | 92 |
| 809-QEAKYETF-816 | 101 | 100 | 99 |
| 834-MTHFRPQLHHSGDM-847 | 88 | 94 | 89 |
| 877-KVSSTSNNL-885 | 89 | 91 | 96 |
| 886-ISTIPSDNL-894 | 101 | 101 | 100 |
| 922-FGKKSSPLTESGGPLSL-938 | 93 | 93 | 96 |
| 922-FGKKSSPLTESGGPLSLSEENNDSKL-947 | 94 | 102 | 99 |
| 939-SEENNDSKL-947 | 97 | 102 | 99 |
| Light chain peptides: |  |  |  |
| 1-EITRTTLQSDQE-12 | 100 | 98 | 97 |
| 1-EITRTTLQSDQEE-13 | 95 | 93 | 96 |
| 25-KKEDFDIYDE-34 | 71 | 88 | 91 |
| 59-WDYGMSSSPHVLRNRAQSGSVPQF-82 | 73 | 79 | 86 |
| 61-YGMSSSPHVLRNRAQSGSVPQFKKVVF-87 | 78 | 86 | 93 |
| 88-QEFTDGSFTQPLYRGEL-104 | 35 | 77 | 93 |
| 90-FTDGSFTQPLYRGEL-104 | 39 | 86 | 94 |
| 90-FTDGSFTQPLYRGELNEHLGLLGPYIRA-117 | 30 | 60 | 83 |
| 90-FTQPLYRGEL-104 | 52 | 82 | 86 |
| 105-NEHLGLLGPYIRAEVED-121 | 3 | 8 | 50 |
| 107-HLGLLGPYIRA-117 | 8 | 10 | 61 |
| 111-LGPYIRA-117 | 7 | 9 | 60 |
| 125-VTFRNQASRPYSF-137 | 8 | 30 | 48 |
| 190-FSDVDLEKDVHSGL-203 | 34 | 34 | 51 |
| 196-EKDVHSGL-203 | 1 | 7 | 27 |
| 196-EKDVHSGLIGPLL-208 | 2 | 31 | 42 |
| 232-FTIFDETKSWY-242 | 2 | 7 | 61 |
| 236-DETKSWYFTENM-247 | 14 | 23 | 41 |
| 248-ERNCRAPCNIQ-258 | 52 | 70 | 91 |
| 265-KENYRFHAING-275 | 17 | 51 | 80 |
| 276-YIMDTLPGL-284 | 2 | 2 | 36 |
| 287-AQDQRIRWYL-296 | 5 | 38 | 65 |
| 304-NIHSIHFSGHVFTVRKKEEYKMAL-327 | 6 | 30 | 73 |
| 313-HVFTVRKKEEYKMAL-327 | 7 | 25 | 43 |
| 336-ETVEMLPSKAGIW-348 | 45 | 77 | 89 |
| 338-VEMLPSKAGIW-348 | 11 | 32 | 29 |
| 354-IGEHLHAGM-362 | 9 | - | 59 |
| 354-IGEHLHAGMSTLF-366 | 3 | 8 | 23 |
| 366-FLVYSNKCQTPLGMASGHIRD-386 | 81 | 89 | 94 |
| 367-LVYSNKCQTPLGMASGHIRD-386 | 38 | 53 | 89 |
| 387-FQITASGQYGQWAPKL-402 | 22 | 34 | 79 |
| 388-QITASGQYGQWAPKL-402 | 26 | 38 | 84 |
| 395-YGQWAPKL-402 | 4 | 10 | 68 |
| 414-WSTKEPFSW-422 | 53 | 78 | 101 |
| 414-WSTKEPFSWIKVDL-427 | 47 | 62 | 80 |
| 423-IKVDLL-428 | 2 | 4 | 56 |
| 429-APMIIHGIKTQGARQKFSSL-448 | 60 | 80 | 92 |
| 469-GNSTGTLMVFFGNVDSSG-486 | 54 | 65 | 89 |
| 476-MVFFGNVDSSGIKHNIFNPPIIAR-499 | 21 | 64 | 80 |
| 476-MVFFGNVDSSGIKHNIFNPPIIARY-500 | 20 | 63 | 77 |
| 477-VFFGNVDSSGIKHNIFNPPIIAR-499 | 24 | 74 | 84 |
| 477-VFFGNVDSSGIKHNIFNPPIIARY-500 | 25 | 68 | 81 |
| 481-NVDSSGIKHNIFNPPIIARY-500 | 22 | 66 | 83 |
| 499-RYIRLHPTHYSIRSTL-514 | 21 | 56 | 85 |
| 533-ESKAISDAQITASSY-547 | 32 | 64 | 80 |
| 552-FATWSPSKARL-562 | 13 | 24 | 57 |
| 585-DFQKTMKVTGVTTQGVKSLL-604 | 60 | 82 | 84 |
| 587-QKTMKVTGVTTQGVKSLL-604 | 73 | 88 | 88 |
| 612-FLISSSQDGHQWTLF-626 | 49 | 57 | 69 |
| 614-ISSSQDGHQ-622 | 69 | 81 | 97 |
| 626-FFQNGKVKVFQGNQDSFTPVVNSLDPPLL-654 | 30 | 67 | 84 |
| 627-FQNGKVKVFQGNQDSFTPVVNSLDPPLL-654 | 30 | 73 | 83 |
| 627-FQNGKVKVFQGNQDSFTPVVNSLDPPLLTRY-657 | 30 | 71 | 84 |
| 642-FTPVVNSLDPPLL-654 | 46 | 62 | 72 |
| 648-SLDPPLLTRY-657 | 10 | 74 | 92 |
| 665-WVHQIAL-671 | 9 | 32 | 33 |

^a^Percentages of deuterium incorporation of 120 peptides after 3 seconds, 2 minutes and 3 hours of incubation are listed. Peptides derived from the B-domain sequence are shown in bold.
B70‑rFVIII, human recombinant factor VIII containing 70% B-domain
